# Functions of exogenous FGF signals in regulation of fibroblast to myofibroblast differentiation and extracellular matrix protein expression

**DOI:** 10.1098/rsob.210356

**Published:** 2022-09-14

**Authors:** Changye Sun, Xiangqin Tian, Yangyang Jia, Mingming Yang, Yong Li, David G. Fernig

**Affiliations:** ^1^ Henan Key Laboratory of Medical Tissue Regeneration, Xinxiang Medical University, Xinxiang, Henan 453003, People's Republic of China; ^2^ Department of Cardiology, Affiliated Zhongda Hospital, School of Medicine, Southeast University, Nanjing, Jiangsu 210009, People's Republic of China; ^3^ Department of Biochemistry, Institute of Systems, Molecular and Integrative Biology, University of Liverpool, Liverpool L69 7ZB, UK

**Keywords:** FGF signalling, TGF-β signalling, fibrosis, ECM proteins, tissue repair, fibroblast activation

## Abstract

Fibroblasts are widely distributed cells found in most tissues and upon tissue injury, they are able to differentiate into myofibroblasts, which express abundant extracellular matrix (ECM) proteins. Overexpression and unordered organization of ECM proteins cause tissue fibrosis in damaged tissue. Fibroblast growth factor (FGF) family proteins are well known to promote angiogenesis and tissue repair, but their activities in fibroblast differentiation and fibrosis have not been systematically reviewed. Here we summarize the effects of FGFs in fibroblast to myofibroblast differentiation and ECM protein expression and discuss the underlying potential regulatory mechanisms, to provide a basis for the clinical application of recombinant FGF protein drugs in treatment of tissue damage.

## Graphical abstract

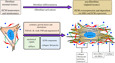

## 1. Introduction

Fibroblasts are widely distributed mesenchymal cells in connective tissue, which regulate embryonic development, homeostasis, and repair of tissues and organs, and are involved in many diseases [1–3]. Fibroblasts are not terminally differentiated cells and they have multi-differentiation potential depending on conditions [1]. In different tissues, fibroblasts display phenotypic diversity [3,4], but in response to tissue injury they normally differentiate into myofibroblasts, a process also called fibroblast activation [5–8]. The myofibroblasts in the damaged tissues overexpress and secrete extracellular matrix (ECM) components to rebuild the tissue [9–11]. However, the excessive synthesis and accumulation of particular types of ECM proteins during wound healing results in tissue fibrosis and scar formation, which seriously affect the physiological functions of the repaired tissues [7,12,13]. Therefore, an understanding of the regulation of the differentiation of fibroblast is critical for the promotion of the functional repair of damaged tissues and limiting fibrosis.

A large body of work demonstrates that fibroblast growth factor (FGF) regulates the functions of various cells and intercellular signalling in the body through binding to its cognate receptor (FGFR), which regulates development, homeostasis, repair of tissues and organs and disease [14–17]. Previous studies found that several numbers of the FGF family play very important roles in cell proliferation, migration, differentiation and survival, which provide significant therapeutic potential for damaged or necrotic tissues [15,18]. Recombinant FGF1 and the low molecular weight isoform of FGF2 (18 kDa, Lo-FGF2, commonly referred to by default as ‘FGF2’) have been used as protein drugs to treat wound healing and scar formation was observed to be reduced [19], and many other FGFs also show great therapeutic effects in repair of various organs [20,21]. Although FGFs have many beneficial functions in tissue repair and regeneration, their activity and mechanism on fibroblast activation and fibrosis are of great concern. In pathological analysis, FGF2 was found to be increased in many damaged tissues [22,23], whereas several FGFs have been shown to inhibit the activation of fibroblasts isolated from multiple organs [21,24,25]. This review aims to provide a brief overview of fibroblast activation mechanism, structure and binding selectivity of FGF and FGFR, the effect and signalling of exogenous FGFs in fibroblast activation and ECM expression, and the perspectives on clinical application of FGF drugs to provide a sound foundation for their clinical translation.

## 2. Fibroblast to myofibroblast differentiation after injury

**Figure 1.**
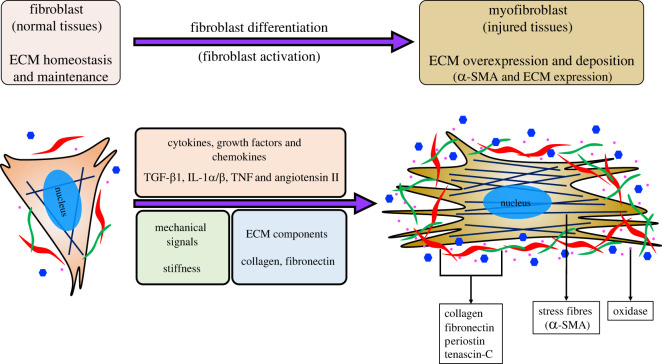
Fibroblast to myofibroblast differentiation. Fibroblast to myofibroblast differentiation, also termed fibroblast activation, can be stimulated by multiple factors. Myofibroblasts express and secrete abundant growth factors, ECM proteins, oxidases and GAGs to contribute to further fibroblast differentiation and ECM deposition.

In normal tissues, fibroblasts express and secrete a large proportion of the soluble effectors, growth factors, cytokines and chemokines, which regulate cell communication and homeostasis [3,9]. After tissue injury, fibroblasts are activated and express abundant alpha-smooth muscle actin (α-SMA), and differentiated into myofibroblasts to secret more ECM proteins (e.g. collagen, elastin and fibronectin) and lysyl oxidase (LOX) [3,26]. ECM remodelling by overexpression, degradation and cross-linking of ECM proteins in the necrotic tissue are direct factors leading to fibrosis tissues (e.g. skin scars, fibrotic liver and lung, renal fibrosis and myocardial damage). There are several potential key factors that drive fibroblast activation: (1) cytokines, growth factors and chemokines; (2) mechanical signals; and (3) ECM and matricellular proteins (figure 1) [1,10].

Inflammatory cytokines and growth factors, such as transforming growth factor-β (TGF-β), interleukin-1α/β (IL-1α/β), tumour necrosis factor-α (TNF-α) and angiotensin II, were found to induce fibroblast activation in injured tissues [10,27]. Of these, TGF-β1, a member of the TGF-β/BMP family, is a principal cytokine, which plays a crucial role in fibroblast activation and the production of ECM proteins and glycosaminoglycans (GAGs) [28–32]. TGF-β1 is secreted as an inactive protein together with latency-associated peptide (LAP) [28]. After the cleavage of the LAP, TGF-β1 is released and activated for binding to its cell-surface TGF-β receptor (TGF-βR) [28]. The type I and II receptors are transmembrane proteins, which can bind to TGF-β1 and then induce the phosphorylation of serine–threonine kinases [28,29]. Smad2 and Smad3 are key downstream signalling proteins, which are phosphorylated by activated TGF-βR and then form a complex with Smad4 to regulate gene expression in the nucleus (e.g. α-SMA and collagens [28]). Deletion of Smad3 reduces collagen deposition in the infarcted heart, indicating the regulation of TGF-β1/Smad signalling on fibrosis [28,29,33,34]. Moreover, TGF-β1 can also activate Smad-independent pathways, in which extracellular signal-regulated kinase (ERK) could also be activated by FGF signalling [29].

Mechanical signals are direct physical stimulations in the body and they also play a role in fibroblast activation. Mechanical signals could change the ECM composition of fibroblast, which would release TGF-β and increase α-SMA expression [35,36]. The *in vitro* work also show that fibroblasts cultured on soft gel express less α-SMA than fibroblasts cultured on hard gel or tissue culture plate [37,38]. ECM proteins, to some extent, could also stimulate fibroblast activation and ECM secretion. A previous finding found a hybrid matrix containing collagen I and collagen III effectively activated fibroblasts and collagen production was in return increased [39]. Reorganization of ECM could change the tissue hardness, which hints that ECM proteins may regulate fibroblast activation by mechanical signals. Although fibroblasts could be activated by multiple factors, tissue injury by chronic disease or damage is always the primary cause and the fibroblast to myofibroblast differentiation requires activation of certain intracellular signalling pathways (e.g. TGF-β signalling); more studies are expected to illustrate the systemic regulation mechanisms that drives fibroblast activation.

## 3. Structure and selectivity of FGF and FGFR binding

FGF family consists of 22 members (FGF1-FGF14, FGF16-FGF23), including four intracellular FGFs (FGF11-FGF14), three endocrine FGFs (FGF19, FGF21 and FGF23) and the remaining 15 paracrine FGFs. Paracrine FGFs regulating the activities of local cells play essential roles in embryonic development and in adult tissues, giving them therapeutic potential in tissue repair.

**Figure 2.**
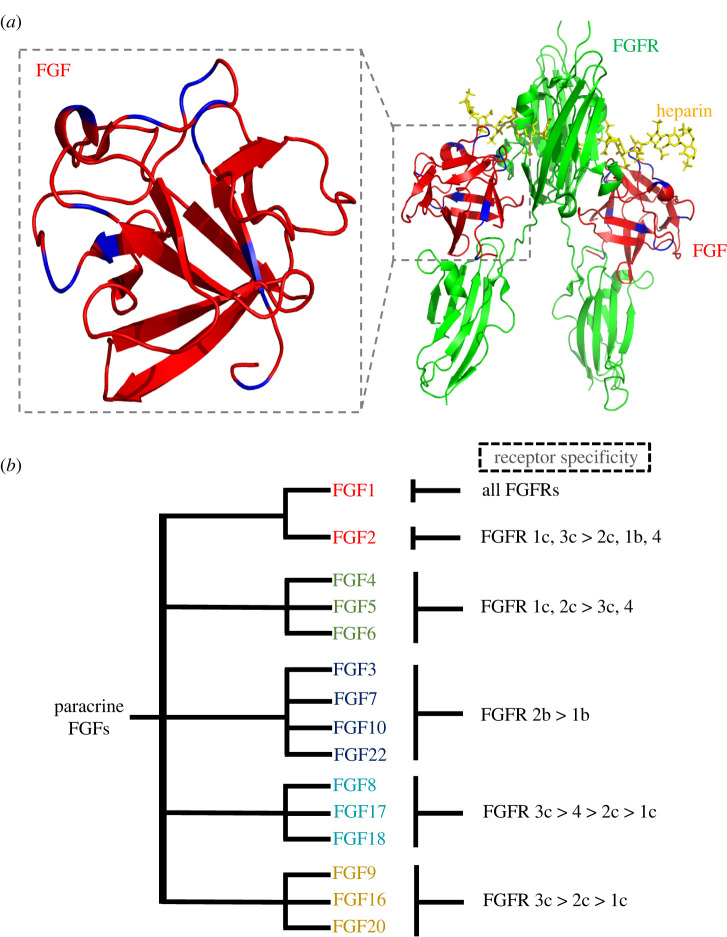
Structure and specificity of FGF-FGFR interactions. (*a*) FGFs interact with the D2 and D3 domains and their linker in FGFR to form a complex with/without heparin/HS (PDB: 1FQ9 [40]). (*b*) Interaction of FGFs and FGFRs shows a degree of specificity, which regulates their biological activities [41].

Owing to the conservation of their peptide sequence, FGFs share a similar core 3-dimensional structure consisting of 12 antiparallel *β* strands that forms a β-trefoil fold (figure 2*a*) [14,40]. FGFRs, which are transmembrane receptors for FGF binding, are the key to transferring induced signals into the cell, which then regulate the target cell activities [15,42,43]. Five different FGFRs (FGFR1–4 and FGFR like 1 (FGFRL1)) and their alternatively spliced isoforms bind FGFs and activate different signalling pathways or the same signalling pathway in different target cells [16,44,45]. FGFR1–4 possess three extracellular immunoglobulin-like loops, I, II and III (often termed D1, D2 and D3), a transmembrane linker and a cytoplasmic tyrosine kinase domain [14,15,44]; FGFRL1 has the same extracellular architecture, but its intracellular domain lacks the tyrosine kinase [45–47]. FGF ligands bind to D2, D3 and their linker in the FGFR, and heparan sulfate (HS) chain could cross-link FGF and FGFR to form a stabilized complex for signal transduction (figure 2*a*).

FGFRs have varying degrees of selectivity for different FGFs, and the selectivity is most conserved for FGFs in the same subfamily (figure 2*b*) [41,48]. Whereas FGF1 is a universal ligand for all the FGF receptors, the other FGFs all exhibit a preference for particular FGFRs and their D3 ‘b’ or ‘c’ isoforms (figure 2*b*) [41]. Of the FGFs, FGF1 and FGF2 with multiple receptor binding affinities possess the ability to regulate the cell functions in most organs. The previous findings found that FGF2 could regulate the activities of fibroblast, epithelium and smooth muscle cell to promote tissue repair, indicating FGF2 has a wide range of therapeutic effects in diverse organs [23,49,50]. Members of FGF7 subfamily (FGF3, FGF7, FGF10 and FGF22) acting on FGFR2b of epithelial cells also have the potential to treat multiple organs by acting directly on the epithelium (e.g. kidney, lung, intestinal tract and skin [51,52]). Members of FGF9 subfamily (FGF9, FGF16 and FGF20) may have therapeutic functions in myocardial repair [53]. FGF6 was found to be specially expressed in skeletal muscle, and may play roles in muscle repair [54]. FGF18, which as sprifermin, was recognized as a promising candidate for treatment of cartilage injury [55]. HS or its experimental proxy heparin enhances the stability of the FGF-FGFR complex to bring about many, though not all biological functions, such as mitogenic activity [56].

## 4. Roles of FGFs in fibroblasts activation of multiple organs

FGFs can promote tissue repair by regulating cell proliferation, survival and angiogenesis. However, their effects in tissue fibrosis regulation remains a concern in the clinical application to treat wound healing, since tissue fibrosis is often related to tissue injury [1,10]. In this section, we summarized the roles of FGFs in fibroblast-to-myofibroblast differentiation in multiple organs to understand their functions in fibrosis.

### 4.1. Heart

After myocardial injury, cardiac fibroblasts (CFs) are induced by TGF-β1 and differentiated into activated myofibroblasts to cause ECM deposition and myocardial fibrosis [25]. FGF family proteins as a potential myocardial repair treatment drug can protect cardiomyocytes and promote vascular regeneration, but the regulatory function and mechanism of FGFs on myocardial fibrosis is an issue in the treatment of myocardial injury [57]. In regulation of CFs, the effects of both low molecular weight Lo-FGF2 (18 kDa) and high molecular weight FGF2 (Hi-FGF2, greater than 20 kDa) were studied. Lo-FGF2 is normally a secreted effector which binds to pericellular HS and FGFRs to induce the intracellular signals, while Hi-FGF2 is mostly transported into cell nucleus after synthesis [58–60]. Different isoforms of FGF2 show distinct effects on fibroblast activation. Several studies found that treatment of CFs with Lo-FGF2 could inhibit TGF-β1-induced α-SMA protein expression and collagen gene expression, and the TGF-β1-induced collagen gel contraction was also reduced [25,61,62]. Kardami's group reported that neutralization of upregulated Hi-FGF2 reduced the accumulation of proteins related to myofibroblast differentiation and fibrosis, including α-SMA, extra-domain A fibronectin and procollagen [62].

**Figure 3.**
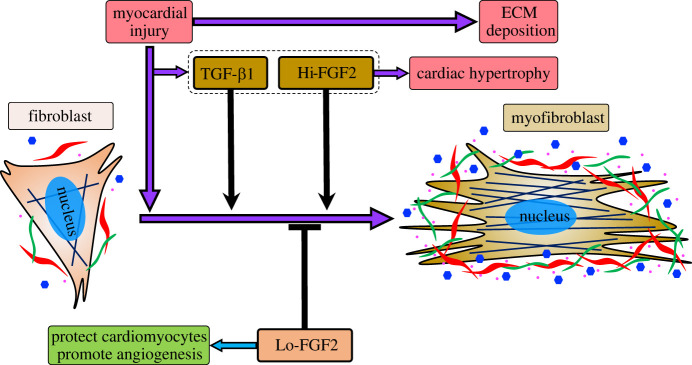
Functions of Hi-FGF2 and Lo-FGF2 in myocardial repair. Hi-FGF2 and Lo-FGF2 show different effects in myocardial repair. Hi-FGF2 induces cardiac hypertrophy and fibroblast activation, while Lo-FGF2 can suppress fibroblast activation, protect cardiomyocytes and promote angiogenesis [18,62,63]. Hi-FGF2: high molecular weight FGF2 (greater than 20 kDa); Lo-FGF2: low molecular weight FGF2 (18 kDa).

Research from Kardami's group in 2007 described the different functions of Lo-FGF2 and Hi-FGF2 in rat myocardial injury [63]. It was found that Lo-FGF2 significantly reduced myocardial cell death and increased the regeneration of small vessels, while the application of Hi-FGF2 caused cardiac hypertrophy (figure 3) [18,63]. These suggest Lo-FGF2 (18 kDa), regulating cell survival, angiogenesis and ECM remodelling, has great potential in myocardial infarction treatment rather than Hi-FGF2. In the following, Lo-FGF2 is referred to as FGF2, since this is the only isoform used to date in experimental and clinical therapeutics.

### 4.2. Lung

A variety of lung diseases result in pulmonary fibrosis, which is characterized by fibroblast proliferation, migration and differentiation to myofibroblasts [21,64]. TGF-β1 is strongly associated with this differentiation, which can be rationalized through TGF-β1 stimulating Smad2 phosphorylation and increasing α-SMA expression [8,21,64]. It was recognized that FGF1 (20 ng ml^−1^) with heparin could increase collagenase expression and reduce collagen expression in primary human lung fibroblasts [24,65,66]. FGF1 with heparin also inhibited TGF-β1-induced Smad2 phosphorylation, α-SMA expression and collagen gel contraction, suggesting FGF1 inhibited myofibroblast differentiation by negative regulation of the TGF-β/Smad signalling axis [24]. Moreover, expression of α-SMA in normal lung fibroblasts was reduced by FGF1 and FGF9, but not FGF18 [67]. It was also found that FGF2 mediated pulmonary fibroblast differentiation and pulmonary fibrosis, potentially though its inhibition of TGF-β-induced phosphorylation of p38 MAPK and c-Jun N-terminal kinase (JNK) [68,69].

In a rat model, overexpression of FGF1 markedly attenuated pulmonary fibrosis induced by TGF-β1 overexpression and expression of α-SMA in lung tissue was also in return reduced [24]. Guzy *et al.* found that knockout of *Fgf2* did not alter mice pulmonary fibrosis induced by bleomycin, but increased deficient recovery of epithelial integrity indicating FGF2 is a protective growth factor after lung injury [23]. Another related study demonstrated that FGF2 decreased bleomycin-induced pulmonary fibrosis by inhibition fibroblast activation and collagen production suggesting FGF2 is an antifibrotic factor [68].

### 4.3. Cornea

Corneal wounding induces the fibroblast to myofibroblast differentiation [8]. Primary corneal fibroblasts were isolated from human corneal rims or rabbit corneas. It was found that TGF-β1 increased the expression of α-SMA, type I collagen (COL I) and type III collagen (COL III), suggesting TGF-β1 activated myofibroblast differentiation [70]. As primary corneal fibroblasts were incubated with FGF1 or FGF2, the expression of α-SMA, TGF-βRs, COL I and COL III and cadherins were reduced [70–72]. At 1 ng ml^−1^ the inhibitory effect of FGF1 was insufficient to prevent such changes, but 10 ng ml^−1^ to 80 ng ml^−1^ FGF1 significantly inhibited α-SMA expression, indicating the inhibitory function of FGF1 is concentration-dependent [70]. The decrease of TGF-βRs regulated by FGF1 or FGF2 shows the negative regulation role of FGF signalling on TGF-β signalling [70].

### 4.4. Skin

Wound contraction is regulated by fibroblast and its differentiated counterpart, the myofibroblast. Both TGF-β and FGF2 are involved in the differentiation of dermal fibroblasts [9,73]. Dermal cells were isolated from porcine skin to determine the regulation effects of TGF-β and FGF2 on the cells [74]. It was found that the expression of TGF-β protein was significantly increased after 4 days incubation *in vitro* and the cells were differentiated into myofibroblast after passaging [74]. Treatment with FGF2 promotes dermal fibroblast phynotype and inhibits its α-SMA expression [73,74].

Moreover, FGF1 and FGF2 were found to suppress TGF-β-induced myofibroblast differentiation of rat hepatic stellate cells and airway and aortic smooth muscle cells, respectively [49,75–77]. These published studies suggest that FGFs have the ability to inhibit the differentiation of fibroblasts of various organs.

The previous work summarized in table 1 shows that FGF2 was mostly studied in the regulation of fibroblast differentiation. Although Hi-FGF2 was recognized as a fibrosis inducer, recombinant FGF2, usually referring to Lo-FGF2, inhibited activation of fibroblasts from different organs and reduced the expression of α-SMA and collagen proteins (table 1). The effect of FGF1 was studied with fibroblasts from lung and cornea and its inhibitory effects were similar to those of FGF2 (table 1). It was found FGF9 could also have an inhibitory effect on lung fibroblasts, but it is weaker than that FGF1 [67]. FGF18 did not show obvious inhibitory effect on lung fibroblast activation [67]. These findings suggest that FGFs may have different degrees of inhibitory functions on fibroblast activation and ECM remodelling.

**Table 1.** Functions of FGFs in fibroblast to myofibroblast differentiation.

organ	ligand (ng ml^−1^)	model (source)	functions
heart	FGF2 (5, 20)	cardiac fibroblast (human atrial and ventricular tissue)	FGF2 reduced TGF-β1-induced collagen gel contraction [25]
			FGF2 reduced TGF-β1-induced α-SMA protein expression and *collagen* gene expression [25]
	Hi-FGF2/Ab-Hi-FGF2 ligand	cardiac fibroblast (human atrial tissue)	neutralization of Hi-FGF2 with antibody significantly reduced expression of proteins (α-SMA, extra-domain A fibronectin, and procollagen) associated with fibroblast to myofibroblast differentiation [62]
lung	FGF1 ± heparin (20)	lung fibroblast (human) and rat pulmonary fibrosis model	FGF1 ± heparin increased collagenase expression, but reduced by 70%–80% the expression of *Collagen 1* mRNA and protein expression, which might have a protective role in avoiding fibrosis during lung repair [65,66]
			FGF1 + heparin induced deoxyribonucleic acid (DNA) synthesis, but significantly reduced cell growth rate [66]
			FGF1 + heparin significantly reduced TGF-β1-induced α-SMA expression and collagen gel contraction [67]
			FGF1 + heparin significantly decreased TGF-β1-induced Smad2 phosphorylation [24,66]
			overexpression of FGF1 prevented the increase of α-SMA induced by overexpression of TGF-β1 [24]
	FGF2 (2 nM)	lung fibroblast (human)	FGF2 inhibited gene expression of *Acta2* (α-SMA), *Collagen 1 and Ctgf*, and FGF2 overexpression reduces bleomycin-induced lung injury [68,69]
	FGF9/FGF18 (20)	lung fibroblast (human)	FGF9 reduced the expression of α-SMA and COL I induced by TGF-β1 in the control cells isolated from cancer patients, but the effect of FGF18 was not significant [67]
			neither FGF9 nor FGF18 significantly prevents the expression of α-SMA and COL I in fibroblasts isolated from idiopathic pulmonary fibrosis patients [67]
cornea	FGF1/FGF2 ± heparin (1, 10, 20, 40, 80)	corneal fibroblasts (human, rabbit)	FGF1(≥ 10 ng ml^−1^)/FGF2(≥ 1 ng ml^−1^) with heparin effectively decreased expression of α-SMA, TGF-βRs and cadherins [70]
			FGF2 (10 ng ml^−1^) decreased expression of COL I and COL III [71]
skin	FGF2 + heparin (0.44)	dermal cells (human, porcine)	FGF2 with heparin significantly decreased expression of α-SMA, similar to the effect of TGF-β antibody [73,74]

## 5. FGF signalling in fibroblast activation

### 5.1. Receptor binding for fibroblast activation

FGFs bind to their receptors with diverse selectivities, which may relate to their effects on fibroblast activation (figure 2*b*). FGF1, FGF2 and FGF9 can strongly stimulate both FGFR1c and FGFR3c, but FGF2 and FGF9 have a near negligible stimulatory effect on FGFR2b [41,78]. FGF18 can strongly stimulate FGFR3c, but its effect on FGFR1c (4.7% of FGF1) is very low [41]. Since FGF1, FGF2 and FGF9, rather than FGF18, could inhibit fibroblast activation, FGFR1 is more likely to be the key FGF receptor to suppress fibroblast differentiation. Pei-Yu Chen *et al.* [79] found that knockdown of FGFR1 using short hairpin RNAs, rather than FGFR3 and FGFR4, caused activation of TGF-β signalling in the endothelium and endothelial-to-mesenchymal transition (EndMT) [79].

FGFR3 partial knockdown also repressed the effect of FGF9 on lung fibroblast differentiation, indicating FGFR3 signal may be able to suppress fibroblast differentiation [67]. However, a recent study found that TGF-β could selectively upregulate expression of FGFR3 and its ligand FGF9, which induced phosphorylation of AKT, p38, ERK and Ca^2+^/calmodulin-dependent protein kinase 2 (CAMK2) to promote fibroblast activation [80]. Thus, FGFR3 displays different suppressive functions in pulmonary and dermal fibroblasts, and more studies are required to uncover its contributions to these regulatory mechanisms.

### 5.2. FGF signalling regulates fibroblast activation

The interaction of FGFs with their receptors induces the dimerization of receptors, which results in the phosphorylation of two tyrosines in the activation loop of the intracellular tyrosine kinase domain. This in turn activates a variety of kinases and their substrates, which initiate the activation of intracellular signalling pathways. These pathways include mitogen-activated protein kinase kinase (MEK), ERK1/2, AKT and phospholipase C*γ* (PLC*γ*), which regulate cell fate and specific cell activities [42,81]. Previous studies suggest HS/heparin is required for most, but not all signalling [52,82,83]. Since there is a great diversity of FGF ligands, FGFR isoforms, HS structure and feedback loops, the understanding of FGF signalling is still far from complete, though the link between the activation of the ERK1/2 pathway and the stimulation of cell division is well established, at least in cultured cells [81].

Various tissue damages caused by ischemia, physical injury and chronic diseases can result in tissue fibrosis in different organ systems suggesting there are common pathogenic pathways for fibrosis. TGF-β can induce the Smad-dependent signalling pathway to activate the specified transcription factors for fibroblast differentiation and fibrotic gene expression [84,85]. And, it was found FGF1 and FGF2 could inhibit phosphorylation of Smad2 to suppress lung fibroblast activation by inducing ERK1/2 signalling [24,68].

**Figure 4.**
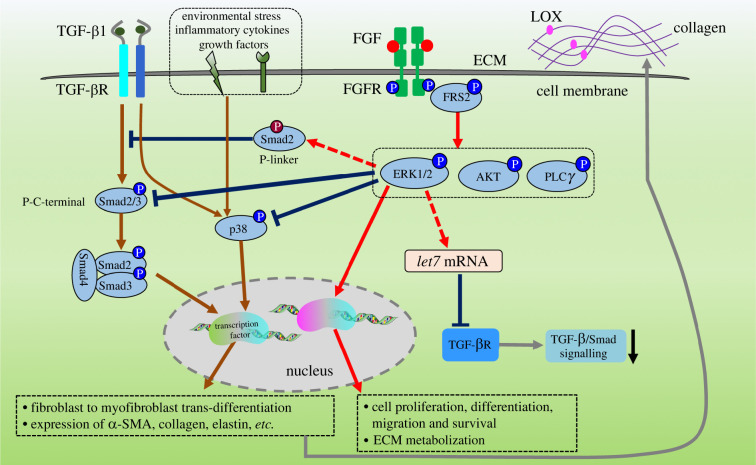
Regulation mechanisms of FGF on fibroblast activation. TGF-β signalling induces C-terminal phosphorylation (P-C-terminal, Ser465/467) of Smad2/3, which then form a complex with Smad4 to regulate fibroblast to myofibroblast differentiation and gene transcription of ECM proteins. Phosphorylation of p38 can be activated by multiple factors, including TGF-β signalling, environmental stress, inflammatory cytokines and certain growth factors, which were also identified to regulate fibroblast differentiation and fibrosis. FGFs can stimulate ERK1/2, AKT and PLC*γ* signalling to regulate various cell functions and metabolization of ECM proteins. ERK1/2 signalling was found to suppress fibroblast activation by inhibition of phosphorylation of Smad2 or p38 [24,68,69]. Moreover, FGF can also inhibit TGF-β signalling by inducing linker phosphorylation (P-linker, Ser245/250/255) of Smad2/3 or decreasing expression of TGF-βR1 labelled with red dashed line [49,86].

Nasreen *et al.* (2005) found that TGF-β1 increased the phosphorylation of p38 MAPK and JNK in pulmonary fibroblasts, and the addition of FGF2 inhibited this phosphorylation [69]. Inhibitors of particular intracellular signalling kinases have been applied to study the regulatory mechanism of FGF2 on tissue fibroblast differentiation. Inhibition of p38 MAPK signalling with small molecular inhibitors has also been shown to reduce fibroblast activation and tissue fibrosis [69], indicating that FGF2 could suppress fibroblast activation by inhibiting p38 MAPK signalling (figure 4). Both PD98059 (inhibitor of MEK1) and U0126 (MEK1/2 inhibitor) can block FGF2-mediated attenuation of fibroblast activation [73,87,88], suggesting activation of MEK1/2-ERK1/2 signalling axis suppresses TGF-β-induced fibroblasts differentiation.

Inflammatory cytokines, environmental stress and some growth factors can also induce phosphorylation of p38 MAPK [27,89,90]. SB202190 and SB203580, p38 MAPK Inhibitors, cannot block the suppressive function of exogenous FGF2 on fibroblast activation, but were sufficient to attenuate TGF-β mediated fibroblast activation [73,87]. Some other p38 MAPK inhibitors (e.g. FR-167653 and SB 239063) also reduced tissue fibrosis, indicating p38 MAPK signalling is involved in cell activation and fibrosis [91,92]. Therefore, p38 signalling is a critical signalling for tissue fibrosis, and FGF-induced MEK1/2-ERK1/2 signalling can inhibit fibroblast activation by downregulation of p38 signalling.

### 5.3. FGF and TGF-β signalling crosstalk

Though multiple signalling pathways were found to be involved in regulation of synthesis of ECM components, TGF-β signalling is the most reported pathway in almost all types of fibrosis. Since FGFs can supress the fibroblast activation induced by TGF-β signalling, the crosstalk between FGF signalling and TGF-β signalling is critical to this regulation. Several crosstalk mechanisms documented in various types of cells are overviewed in the following content to supply a reference for study the regulatory mechanisms of FGFs on fibroblast activation.

The previous studies found that knockdown of the key FGFR adaptor protein, FGF receptor substrate 2 *α* (FRS2*α*) or FGFR1 itself increased the phosphorylation level of Smad2 and also increased gene expressions of TGF-β1, TGF-β2, TGF-βR1, α-SMA and collagens in smooth muscle cells and endothelial cells [49,79]. It was also found that FGFR1 could promote the expression of *let-7* miRNA and overexpression of *let-7* miRNA downregulates the expression of TGF-βR1 (figure 4) [49]. Another study found constitutively active FGFR1 or FRS2*α* by mutation or FGF2 could inhibit the expression of TGF-βR1 and phosphorylation of Smad2 [93]. Moreover, FGF1 and FGF2 were found to reduce the expression of TGF-βR in corneal fibroblasts [70]. Thus, FGF signalling could downregulate TGF-β signalling by inhibiting the expression of TGF-β and TGF-βR proteins (figure 4).

Another crosstalk mechanism is by regulating the phosphorylation of Smad2. Taeko *et al*. (2014) found FGF2, rather than VEGF, EGF and IGF, could supress lymphatic endothelial cell differentiation [86]. And, overexpression of H-Ras, a downstream signalling protein of FGFRs, induced linker-phosphorylated Smad2 which in return supressed C-terminally phosphorylation of Smad2 and inhibited Smad signalling induced lymphatic endothelial cell differentiation (figure 4) [86]. MEK inhibitors (U0126 and PD184352) supressed phosphorylation of ERK1/2, whereas the phosphorylation of C-terminal of Smad2 induced by TGF-β was strongly increased [86]. Another report found that oncogenic Ras could downregulate TGF-β signalling by causing Smad2/3 phosphorylation in the linker region and nuclear accumulation was reduced [94]. Together these data suggest FGF2 could also inhibit TGF-β signalling by inducing Ras-ERK signalling pathway and stimulating linker phosphorylation of Smad2/3 (figure 4).

## 6. Perspectives on clinical application of FGF drugs

In the past decades, some paracrine FGFs have been developed into drugs for clinical treatment or clinical trial [19]. Recombinant proteins of FGF1 and FGF2 have been used to treat skin wound healing (e.g. burns and some surgical wounds) in China [19]. Recombinant FGF2 was first used in human clinical trials in 1992 in the USA, and it was found that the recombinant FGF2 was safe and achieved a greater healing effect with increases in fibroblasts and in capillaries [95]. Subsequently, a large number of clinical trials found that application of recombinant FGF2 drug could reduce the healing time and improve the scar quality [19]. FGF1 was applied to deep partial-thickness burns and skin graft donor site since 2007 in China, and the findings suggest that recombinant FGF1 can accelerate wound healing [96]. FGF18 was used to treat osteoarthritis, a cartilage injury disease, though the study was terminated due to low recruitment [55,97,98]. Currently, there are still some clinical trials to be processed, for example, a clinical study of Kangfuxin (a clinically approved extract from *Periplaneta americana*) and FGF2 in promoting the healing of donor site and FGF1 for the treatment of coronary heart disease [99,100]. Animal experiments also suggest FGFs have great potentials in aiding the repair of various tissues. For example, FGF10 was found to regulate the tissue repair and regeneration in kidney injury [101]. The effects of FGFs in cancers and tissue fibrosis are two critical points of concern in clinical application, which limits their transition to clinical application. As described in a recent review, there is no link between FGF ligands and cancer, since FGF ligands are not oncogenic in themselves and their involvement in cancers is solely due to mutations of the FGFRs [19]. Nevertheless, there are still many issues limiting the clinical efficacy of FGFs, which are discussed in the following content.

### 6.1. Isoforms of FGFs

Due to either alternative translation initiation codons (FGF2, FGF3) or alternative splicing of the mRNA (FGF8), different isoforms of these FGFs are produced, which possess distinct biological functions, for example those of Lo-FGF2 and Hi-FGF2 in cardiac fibroblast activation and in myocardial repair [60,102]. The previous studies demonstrate that Lo-FGF2, rather than Hi-FGF2 is the better candidate for the stimulation of tissue repair [18,63].

### 6.2. Concentration of FGFs

At least some FGFs are documented in development to elicit different cell responses depending on their concentration, reflecting their fundamental functions as morphogens [103,104]. In cultured cells similar concentration-dependent responses are seen and, moreover, different optimum concentrations are measured for cellular responses. For example, in rat mammary fibroblasts (Rama 27), max DNA synthesis was stimulated by 0.3 ng ml^−1^ FGF2, while the stimulation effect became very low as supramaximal concentrations (≥100 ng ml^−1^) were applied [105]. In the previous studies, 10–20 ng ml^−1^ FGF1 and FGF2 concentrations were mostly applied for fibrosis inhibition, but 0.44 ng ml^−1^ FGF2 could significantly inhibit porcine dermal cell activation (table 1). So, the concentration range for the essential biological activity and safety is still not clearly identified. The question related to concentration is the frequency of administration, since FGFs accumulate in ECM, and diffuse by reversible binding to HS [106]. Consequently, the control of the *in situ* concentration of injected FGFs is also a challenging problem.

### 6.3. Stabilization of FGFs with polysaccharides

HS is a sulfated polysaccharide covalently linked to membrane and ECM proteins. The paracrine FGFs are largely HS-dependent since much of their signalling depends on the formation of a ternary complex with the polysaccharide and the FGFR (figure 2*a*). Binding to HS also controls the diffusion of these growth factors and, importantly from a therapeutical perspective, increases their stability towards denaturation and proteolysis. Heparin, an experimental proxy for HS has been used to stabilize FGFs [17,50,107]. In the fibrosis treatment studies, both FGF protein alone and FGF with heparin could supress fibroblast activation and fibrosis-related gene expression, indicating heparin is not a compulsory component supplemented to FGFs for inhibiting fibrosis signalling [66,70]. However, though considered to be a useful supplement to increase the stability and activity of the FGFs, it may also increase their radios of diffusion and more approaches more sophisticated than soluble polysaccharide may be warranted.

### 6.4. Efficient delivery of FGFs

In tissue repair and regeneration, FGF protein drugs are delivered directly to the targeted tissues. However, FGFs are biodegradable molecules and possess certain diffusion ability in the local tissue [108,109]. For example, FGF2 locally administrated into lung was not detected after 2 days [110]. Therefore, an efficient method to control the release of FGF protein drugs is required for an effective therapy. Building on the interaction of FGFs with HS, it was found that several sulfated polysaccharides that are common biomaterials can interact with FGFs (e.g. heparin, dextran sulfate and λ-carrageenan [50,111]). A biomaterial for efficient FGF binding and release is likely to be a preferred means to deliver the desired concentration of FGFs for effective therapy with minimal invasiveness.

## 7. Conclusion

A large body of evidence indicates FGF1 and FGF2 in particular can elicit signals in fibroblasts of various organs that inhibit their differentiation into myofibroblast *in vitro*. This suggests that FGF1 and FGF2 are useful drugs for relieving tissue fibrosis in tissue repair and may underlie at least some of the clinical success in their application [19]. Despite current clinical data demonstrating the potential of FGFs and the major socioeconomic burden caused by tissue fibrosis and insufficient repair, there is still no consensus on how to employ FGFs. This probably arises from weaknesses in the approaches used: they follow candidate signalling pathways, rather than the entire signalling networks, this despite the fact that cultured cells lend themselves to systems analyses and moreover do not follow up with work in animal models and then clinical samples. As a result, we have not fully revealed the function and mechanism of FGF regulating tissue repair.

Genomic and proteomic analyses would enhance our understanding of the subtleties of the effects of these FGFs on fibroblasts and their interaction with those of TGF-β signalling. Moreover, such work would provide a strong foundation for the analysis of preclinical models and, given progress in single cell transcriptomics and proteomics, of clinical samples. Collectively, this work would provide an ensemble of data from which predictive models could be built and so a tailoring of therapy.

## Data Availability

This article has no additional data.
